# The Impact of Oral-Gut Inflammation in Cerebral Palsy

**DOI:** 10.3389/fimmu.2021.619262

**Published:** 2021-02-25

**Authors:** Ana Cristina Fernandes Maria Ferreira, Ryan J. Eveloff, Marcelo Freire, Maria Teresa Botti Rodrigues Santos

**Affiliations:** ^1^Postgraduate Program in Dentistry, Department of Individuals With Special Needs, Cruzeiro do Sul University, São Paulo, Brazil; ^2^Department of Genomic Medicine and Infectious Diseases, J. Craig Venter Institute, La Jolla, CA, United States; ^3^Department of Infectious Diseases, School of Medicine, University of California, San Diego, La Jolla, CA, United States; ^4^Department of Dentistry, Association for Assistance to Disabled Children, São Paulo, Brazil

**Keywords:** cerebral palsy, inflammation, cytokines, quality of life, caregiver priorities and child health index of life with disabilities, constipation, antiepileptic drug

## Abstract

**Background:** Oral-gut inflammation has an impact on overall health, placing subjects at risk to acquire chronic conditions and infections. Due to neuromotor disturbances, and medication intake, cerebral palsy (CP) subjects present intestinal constipation, impacting their quality of life (QOL). We aimed to investigate how oral inflammatory levels predicted gut phenotypes and response to therapy.

**Methods:** A total of 93 subjects aging from 5 to 17 years were included in the study, and assigned into one of the 4 groups: CP with constipation (G1, *n* = 30), CP without constipation (G2, *n* = 33), and controls without CP with constipation (G3, *n* = 07) and without CP and without constipation (G4, *n* = 23). In addition to characterizing subjects' clinical demographics, medication intake, disease severity levels, salivary cytokine levels [TNF-α, interleukin (IL)-1β, IL-6, IL-8, IL-10], and Caregiver Priorities and Child Health Index of Life with Disabilities (CPCHILD). Statistical significance was evaluated by Shapiro-Wilks, Student's *T*-Test, ANOVA, and ANCOVA analysis.

**Results:** Salivary proinflammatory cytokines were highly correlated with the severe form of gut constipation in G1 (*P* < 0.001), and out of all cytokines IL-1β levels demonstrated highest correlation with all gut constipation *(P* < 0.05). A significant relationship was found between the type of medication, in which subjects taking Gamma-Aminobutyric Acid (GABA) and GABA+ (GABA in association with other medication) were more likely to be constipated than the other groups (*P* < 0.01). Cleary salivary inflammatory levels and gut constipation were correlated, and impacted QOL of CP subjects. G1 presented a lower QOL mean score of CPCHILD (49.0 ± 13.1) compared to G2 (71.5 ± 16.7), when compared to G3 (88.9 ± 7.5), and G4 (95.5 ± 5.0) (*P* < 0.01). We accounted for gingival bleeding as a cofounder of oral inflammation, and here were no differences among groups regarding gender (*P* = 0.332) and age (*P* = 0.292).

**Conclusions:** Collectively, the results suggest that saliva inflammatory levels were linked to gut constipation, and that the clinical impact of medications that controlled gut was reliably monitored via oral cytokine levels, providing reliable and non-invasive information in precision diagnostics.

## Introduction

Cerebral palsy (CP) is a life-limiting and costly disability characterized by a permanent neuromotor disorder affecting movement and by non-progressive degeneration of the brain (1, 2). As the major etiological factor for severe disability, the estimated prevalence of CP ranges from 2.3 to 2.9 per 1,000 live births observed in the United States (2011–2012 National Survey of Children's Health and the 2011–2013 National Health Interview Survey) (3). Children with CP experience limitations to everyday activities, adversely influencing their quality of life (QOL). CP can be accompanied by difficulties related to perception, sensation, behavior, cognition, communication, as well as epilepsy. Furthermore, CP subjects' QOL can be impacted by the development and progression of musculoskeletal disturbances such as oral-gut motor impairment through muscle spasticity (1).

Due to an abnormal increase in muscle tone and injury to neural pathways, spasticity acts as a negative factor in the lives of 85–90% of subjects with CP (1, 4, 5), impacting understanding subjects overall quality of life. The progression and severity of movement disability is classified by the Gross Motor Function Classification System (GMFCS) (6). GMFCS is stratified into five levels of body mobility: Level I (walking without limitations), Level II (walking with limitations), Level III (walking using a hand-held mobility device), Level IV (self-mobility with limitations), and Level V (transported in a wheelchair) (6). Our previous studies demonstrated that out of 254 subjects, 50 (19.7%) had GMFCS I, II, or III while 204 (80.3%) presented GMFCS IV or V (7–9). Notably, epilepsy prevalence increases in spastic CP subjects at the highest GMFCS classifications (IV and V) (10). Thus, there is an imminent need to discover non-invasive and precise diagnostics to predict these phenotypes seen in CP.

Gut constipation and lack of gastrointestinal control is highly prevalent in CP subjects, varying from 25 to 74% (3). Constipation is caused by multiple factors including: (1) reduced intake of fiber and liquids (responsible for the digestive system functioning) (11), (2) central nervous system damage (12), (3) mobility reduction (13), (4) and/or the use of antiepileptic drugs (AEDs) (11, 14). In subjects using AEDs the incidence of gut constipation is elevated (7). Other side effects co-occurring with gastrointestinal complications include oral dysbiosis, gingival bleeding (GB), and an increase of systemic inflammation (15). GB is found at a high frequency that may be due to the same factors that predispose to tooth decay and lead to the accumulation of biofilm (16). Difficulties in performing daily oral hygiene, intraoral sensitivity and orofacial motor dysfunction are the main contributing factors (17). Because epilepsy affects 77% of the subjects with CP, and standard clinical treatment for epileptic CP subjects is based on therapy with antiepileptic drugs (AEDs) (18). As the principal inhibitory neurotransmitter of the central nervous system and also a regulatory signal for muscle tone, Gamma-Aminobutyric Acid (GABA) is used for neuronal excitability control (e.g.,: benzodiazepines, phenobarbital, topiramate, vigabatrin, gabapentin enacarbil, and ezogabine). Side effects of AEDs can range from subjective reports of mild drowsiness to life-threatening neurologic and gastrointestinal consequences (19).

In addition to motor disturbances, CP subjects present impairment in inflammatory pathways. Epidemiological studies on premature births correlate the presence of high inflammatory levels in the umbilical cord, amniotic fluid, and fetal blood with white matter injury. In fact, premature babies are born in a state of severe inflammation (20–22). While CP and developmental impairment have diverse etiology, at the center of disease development, impaired inflammation regulates CP clinical phenotypes. Recently, attributed to dysregulated cytokine production, CP inflammation is modulated mainly by diet restrictions, gut dysfunction, medication intake. In epileptic subjects, seizures alone are shown to stimulate the synthesis of pro-inflammatory and pro-convulsive cytokines (23). IL-1β is a mediator of inflammatory response, cell death by apoptosis and regulator of bone resorption. This is a central cytokine modulated by master gene, such as inflammasome regulates how the host response is shaped in chronic conditions, including gut and oral dysbiosis, such as inflammatory bowel disease and periodontitis (24). In addition, other factors such as TNF-α correlate have shown synergistic actions with IL-1β, especially when in exacerbation response (25). While IL-6 levels have also been found in the crevicular gingival fluid in disease subjects (26), a chemokine interleukin-8 (IL-8 or CXCL8) is key migrating agent for myeloid-derived cells, such as neutrophils (27). In contrast, to potent activators of inflammation, several molecules counteract their actions. Central to IL-1β, feedback look is the production of interleukin-10 (IL-10) in a timely manner to spatially regulate tissues to return to homeostasis by potent actions (28). Inflammatory levels are the target of direct actions of drugs, and as side effects. AEDs can influence the immune system by modifying interleukin and chemokine concentrations in blood; these changes seem to be independent of the serum concentrations of these drugs (29, 30). In CP investigations, cytokine levels have been less investigated, especially in the context to their comprehensive clinical phenotypes, and in the context of response to essential therapeutics, which is important to their survival.

Here we investigated how oral inflammatory signatures correlated with gut constipation and impacted the QOL of each subjects. Our findings indicated significant correlations between proinflammatory cytokine compositional changes detected in saliva of CP patients and gut constipation, and medication intake. The specific correlation of severe forms of gut constipation with IL-1β cytokine was specific and did not include other inflammatory factors significantly, was confirmed by cofounder evaluations. While we did not include dental plaque, we accounted for gingival bleeding as a cofounder of oral inflammation, and there were no differences among groups regarding gender, and age. In addition, we assayed all clinical factors that impacted the patient's QOL and each cytokine level. Clearly, medication intake was a clinical factor with direct impact to gut constipation and consequently QOL. While our investigation cannot determine causation of clinical phenomic factors that were impacted by AEDs, including dental plaque or medication biochemical actions, positive correlation by host salivary inflammatory levels was specific for certain types of cytokines and not all the molecules assays, demonstrating the direction of future research. We propose that this data reveals a much greater importance of salivary cytokines for evaluation of systemic health and their gut response to various clinical challenges. Here, we aimed to investigate how salivary inflammatory levels predicted intestinal phenotypes. Because inflammation impacts the overall subject, we further investigated how drug intake and clinical factors control overall QOL of subjects with CP. Altogether, the results suggest that saliva inflammatory levels were linked to gut constipation, an innovative and non-invasive method to monitor QOL, improving precision medicine and dentistry.

## Materials and Methods

### Study Design

This study was reviewed and approved by the Research Ethics Committee of the Cruzeiro do Sul University-Brazil Platform, São Paulo, Brazil (IRB #2,452,626). Written informed consent was obtained from the guardian of each child or adolescent after they were informed about the study. A cross-sectional study was performed with subjects with spastic CP diagnosis, who received a physical rehabilitation treatment at a referral center in São Paulo, Brazil, at the time of data collection.

### Participants

Seventy subjects with a medical diagnosis of CP were invited to participate in this study at Disabled Child Care Association (AACD) in São Paulo, Brazil. Inclusion criteria were a medical diagnosis of spastic CP ranging from 5 to 17 years, both male and female, and the presence or absence of constipation. Subjects who presented progressive or neurodegenerative lesions or uncooperative behavior were excluded during clinical oral examinations. Seven subjects with CP were excluded from the research because they did not collaborate during the oral exams. The control group consisted of 30 normotypic subjects who attended the Pediatric Dentistry Clinic of the Faculty of Dentistry of the Cruzeiro do Sul University, residing in the same health district as the study group (that is, with the same health practices developed in health services), at the time of data collection. The final sample of the study was composed of subjects assigned into one of the 4 groups (G1-4) based on the prevalence of (1) CP and (2) Constipation. Subjects were assigned as CP with constipation (G1, *n* = 30), CP without constipation (G2, *n* = 33), and controls without CP with constipation (G3, *n* = 07) and without CP and without constipation (G4, *n* = 23). Both demographic and clinical data were collected for each subject from the year 2018 until 2019. Data regarding gender, age, race (white, black and others), caregiver occupation and education, family income, the medical diagnosis of CP according to the type of movement disorder (spastic), clinical pattern: tetraplegia (a system-wide decay of neuromotor function, represented by increased muscle tone in all four limbs and trunk involvement); diplegia (increased muscle tone in lower limbs, but may affect upper limbs but to a lesser extent), and hemiplegia (or increased muscle tone in a hemibody), furthermore GMFCS (levels I–V) (6) and use of AEDs were collected from their medical records. Caregivers answered the Caregiver Priorities and Child Health Index of Life with Disabilities (CPCHILD) (31) that consists of 36 items rated in six sections that are scaled from 0 (worst) to 100 (best) and were averaged to determine overall QOL. CPCHILD is represented by the following domains: (1) Personal Care (eight items); (2) Positioning, Transfer, and Mobility (eight items); (3) Communication and Social Interaction (seven items); (4) Comfort, Emotions, and Behavior (nine items); (5) Health (three items); and (6) Overall Quality Of Life (one item).

### Gastrointestinal Constipation

This study adopted the clinical constipation definition proposed by the Bristol Stool Scale (BSS) for constipation (32). The translation and adaptation of the BSS into Brazilian Portuguese showed high reliability, indicating its usefulness in the practical clinic for the purpose for which it was planned, both for use in children (33) and adults (34).

### Saliva Collection

Unstimulated whole saliva samples were collected in dental assessment sessions. Subjects were asked to refrain from eating, drinking liquids, or brushing their teeth for at least 1 h prior to saliva collection. The collection was performed with the subjects sitting comfortably in a bright and ventilated room. Whole saliva was collected by passive flow for 5 min. After collection, the Salivette® was centrifuged at 5,000 rpm for 5 min at 4°C (Hettich Centrifuge, model Universal 320R, Tuttlingen, Germany) and frozen in a freezer at −80°C.

### Biomarker Sub-analysis

For a subset of 37 participants, all of whom were CP subjects, we analyzed the salivary cytokines IL-1β, IL-6, IL-8, IL-10, and TNF-α. The analysis of cytokines in saliva was performed using a CBA Cytokine Inflammatory Kit (Becton Dickinson, CA, USA) for the detection of TNF-α, IL-1β, IL-6, IL-8, IL-10. All analyses were performed in duplicate. Briefly, 25μL of fluorescent particles conjugated to antibodies specific for each cytokine were added to 25 μL of the saliva and incubated for 1 h at room temperature away from light. Subsequently, 25 μL of the secondary antibody conjugated to a fluorochrome was added to the mixture and incubated for 2 h at room temperature. The results were compared to a standard curve with serially diluted cytokines. The particles were washed to remove unbound antibodies, resuspended in the wash buffer, and analyzed using a BD Accuri (BD Biosciences). Data acquisition was performed using BD-Accuri C6 Software, and concentrations were determined using FCAP software v.3.0 (BD Biosciences).

### Gingival Index

The evaluation of oral dysbiosis was assayed through the GI (35) by using a millimeter plastic periodontal probe (HuFriedy's Colorvue PerioScreen Kit probe, Chicago, IL, USA), which was gently passed in the gingival margin of all teeth, in reference to the distobuccal papilla, the buccal margin, the mesiobuccal papilla, and the lingual/palatine margin. Partially erupted teeth and residual roots were excluded without replacement. The index was calculated by the percentage of the sum of the subjects values of each tooth divided by the number of faces examined. Classified as positive for gingivitis were the subjects that presented gingival marginal bleeding more than 10% of the total sites evaluated (36). Gingival index was included as a surrogate for oral hygiene.

### Statistical Analyses

Analyses of descriptive statistics were performed to characterize the sample, calculate measures of central tendency and variability for the quantitative variables. The normality assumption of the quantitative variables was evaluated using the Shapiro-Wilks test. When normal distribution was observed, parametric tests were performed. Otherwise, non-parametric tests were selected to determine the significance of intergroups differences. Student's *T*-Test was used for any comparison of two groups (including intergender groups and comparisons of constipated and non-constipated subjects), while two-tailed ANOVA (comparison without covariance correction) and ANCOVA (comparison with covariance correction) were used in comparisons of experimental group, age, cytokine measures, and gingival index. IBM SPSS Statistics (SPSS for Windows, Version 20.0, Armonk, NY: IBM Corp.) and RStudio were used for analyses, with significance thresholds at *P* < 0.05, *P* < 0.01, *P* < 0.001, and *P* < 0.0001. Data was visualized using RStudio with the ggplot2 package and assisted by the dplyr and tidyr packages.

## Results

### Demographics

The sample power was calculated using means and standard deviations of overall domains among G1 (51.0 ± 13.1), G2 (28.5 ± 16.7), G3 (11.1 ± 7.5) and G4 (4.5 ± 5.0) (37). The results showed that the G^*^Power at the 95% confidence interval was 96.88%.

To predict salivary cytokine levels with gut phenotype, we first investigated how overall clinical factors impacted both oral and gut phenotypes in CP subjects. We collected subjects detailed information including demographics, clinical factors, and AEDs as potential influencers of a subject's inflammation, wellbeing, and QOL (Table 1). In our population, 47 of our 93 subjects were female and 46 were male, accurately representing the relatively equal ratio of female to male.

**Table 1:** Population-wide demographics table.

	**G1 - constipated CP^a^**	**G2-No constipated CP^a^**	**G3- constipated control^a^**	**G4- no constipated control^a^**	*p*-value*
**DEMOGRAPHICS**					
Total Subjects	30 (32.25%)	33 (35.48%)	7 (7.52%)	23 (24.73%)	
**Gender**					
Female	19 (66.3%)	14 (42.4%)	4 (57.1%)	10 (43.5%)	0.332
Male	11 (36.7%)	19 (57.6%)	3 (42.9%)	13 (56.5%)	
Age (mean±SD)	8.5 ± 4.1	10.3 ± 3.7	8.8 ± 4.5	10.2 ± 4.3	0.291
**Race**					
White	19 (63.4%)	15 (45.5%)	6 (85.7%)	18 (78.3%)	
Others	9 (30%)	16 (48.5%)	0 (0%)	5 (21.7%)	0.08
Black	2 (6.4%)	2 (6%)	1 (14.3%)	0 (0%)	
**Occupation (Caregiver)**					
Employed	11 (36.66%)	13 (39.39%)	5 (71.42%)	19 (82.60)	0.0017*
**Education**					
≤8 years	21 (70%)	23 (69.69%)	4 (57.15%)	14 (60.86%)	0.5628
>8 years	9 (30%)	10 (30.31%)	3 (42.85%)	9 (39.14%)	
**Family (income)**					
≥3MW	4 (13.34%)	9 (27.28%)	3 (42.86%)	11 (47.82%)	0.018*
<3MW	26 (86.66%)	24 (72.72%)	4 (57.14%)	12 (52.17%)	
**ORAL HEALTH- PERIODONTAL PARAMETERS**					
Gingival bleeding	32.4 ± 19.8	4.1 ± 3.5	0.6 ± 1.4	0.1 ± 0.6	<0.001*
**Gingivitis**					
Presence	23 (76.7%)	2 (6.1%)	0 (0%)	0 (0%)	<0.001*
Absence	7 (23.3%)	31 (93.9%)	7 (100%)	23 (100%)	
**SYSTEMIC HEALTH- CEREBRAL PALSY**					
**Clinical pattern**					
Tetraplegia	20 (66.66%)	9 (27.27%)	0 (0%)	0 (0%)	0.0009*
Diplegia	10 (33.33%)	15 (45.45%)	0 (0%)	0 (0%)	
Hemiplegia	0 (0%)	9 (27.27%)	0 (0%)	0 (0%)	
**GMFCS**					
I - II – III	0 (0%)	14 (42.4%)	not applicable	not applicable	<0.0001*
IV – V	30 (100%)	19 (57.6%)	not applicable	not applicable	
**Medication**					
GABA	5 (16.7%)	1 (3%)	0 (0%)	0 (0%)	
GABA+	9 (30%)	2 (6.1%)	0 (0%)	0 (0%)	
Sodium	5 (16.7%)	4 (12.1%)	0 (0%)	0 (0%)	0.0063*
Calcium	1 (3.3%)	1 (3%)	0 (0%)	0 (0%)	
No medication	10 (33.3%)	25 (75.8)	7 (100%)	23 (100%)	
**Quality of life**					
Personal care	20.2 ± 21.6A	53.6 ± 37.5B	80.0 ± 23.8C	96.2 ± 8.4C	<0.05*
Positioning, transfer and mobility	29.3 ± 26.1A	57.9 ± 34.4B	100.0 ± 0.0C	99.9 ± 0.5C	<0.05*
Communication and social interaction	55.6 ± 28.3A	82.1 ± 19.3B	100.0 ± 0.0C	100.0 ± 0.0C	<0.05*
Comfort, emotions and behavior	55.6 ± 22.7A	81.2 ± 16.00B	91.3 ± 12.8C	96.1 ± 4.9C	<0.05*
Health	73.6 ± 18.2A	83.8 ± 12.9B	90.3 ± 10.2C	96.5 ± 5.7C	<0.05*
Overall quality of life	55.7 ± 24.3A	70.3± 15.2B	71.4 ± 19.5C	81.7 ± 20.8C	<0.05*
Domains means	49.0 ± 13.1A	71.5 ± 16.7B	88.9 ± 7.5C	95.5 ± 5.0C	<0.05*

a *Values represent mean ± SD or percentage (%)*;

* *p-values for group comparisons were significant at 0.05; Chi-square, ANOVA, One-Way, Kruskal-Wallis tests. Different letters denote statistically significant differences (p < 0.05). Uppercase letters compare values horizontally (intragroup evaluation). SD, standard deviation; BOP, Bleeding on probing; MW, minimum wage, unit for measuring income in Brazil, broadly corresponding to $251 U.S. during the study period*.

*Numbers and relevant statistics for study groups. Groups are defined as G1 (CP with constipation), G2 (CP without constipation), G3 (control with constipation), and G4 (control without gut constipation)*.

G1 was composed of 30 non-ambulatory subjects (GMFCS levels IV and V) and G2 of 14 ambulatory subjects (GMFCS levels I, II, and III) (*P* < 0.0001). Lower QOL was observed for constipated subjects presenting the clinical pattern tetraplegia when compared to non-constipated CP (*P* < 0.001) (Table 1). Interestingly, gender was significantly associated with constipation (*P* < 0.05) with females appearing more likely to be constipated in our population. There were no significant differences among groups regarding gender (*P* = 0.332), age (*P* = 0.291), race (*P* = 0.08), and education (*P* = 0.5628); however, significant differences were found on the basis of caregiver occupation (*P* < 0.01) presenting the caregivers CP group a higher number of unemployed, and lower family income (*P* < 0.05).

**Figure 1:**
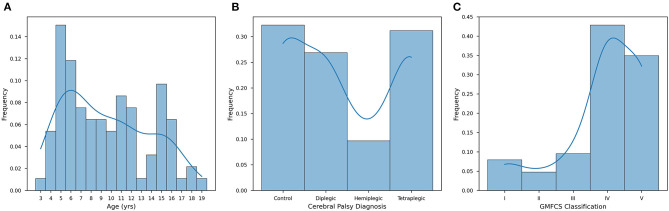
Distribution patterns of the CP study population. **(A)** Individuals from the cohort aged from 3 to 19, with a mean age of 9.57 ± 4.20 years (*n* = 93). **(B)** The study population was composed of healthy individuals (controls, 32.26%), tetraplegics (32.26%), diplegics (26.88%), and hemiplegics (9.68%). **(C)** Distribution is measured along the GMFCS scale from I-V for only G1 and G2. Stages I-III are considered ambulatory, while stages IV and V require significant assistance (typically a wheelchair or mobility scooter). Stages I, II, and III are less represented (7.94%, 4.76%, and 9.52%, respectively), while stages IV (42.86%) and V (34.92%) make up the vast majority of G1 and G2 (*n* = 93). For each of these histograms, an LM regression is overlaid to make visual trend observation more convenient.

Distribution of age in our population shows a peak at approximately 6 years of age, with a slight steady dropoff in population representation until approximately age 14, with a steep dropoff in population representation from ages 14–19 (Figure 1A). It is important to note once more that the GMFCS levels are dependent on CP diagnosis, as tetraplegia, diplegia, and hemiplegia insinuate a certain level of mobility reduction (Pearson *R* = 0.787). We found this correlation to be pertinent in further analyses of GB, salivary cytokines, and QOL.

### CP Diagnosis

When diagnosing CP, there are three widely accepted presentations: (1) hemiplegic; (2) diplegic; and (3) tetraplegic. In our population, the distribution of subjects is made up of these three groups as well as healthy subjects (control). In our population 9.68% of subjects were hemiplegic, 26.88% diplegic, 31.18% tetraplegic, and 32.26% normotypic (Figure 1B). CP diagnosis was found to be significant in our population (*P* < 0.001) in determining QOL (Table 1). Another datapoint we used to determine the severity of a patient's condition was motor function ability as classified by the GMFCS scale, which rates a child's movement from I (walking without limitations) to V (total motor dependence). The GMFCS on our population, levels IV (29.03%) and V (34.92%) make up the majority of our population and are reserved for subjects with clinically advanced CP, namely diplegic and tetraplegic CP (Figure 1C). We found that a subject's GMFCS level was statistically significant in determining their QOL (*P* < 0.001). This significance reflects the impact the severity of cortex injury on functional motor independence; diplegic and quadriplegic subjects are limited exclusively to GMFCS IV and V and have a significant motor impairment that requires powered mobility assistance and/or a physically able caretaker.

### Constipation

Indirect symptoms of CP are not limited to the oral cavity: of our 63 subjects who presented CP, 30 were constipated at the time of examination, representing a much higher prevalence of constipation (47.62%) compared to the estimated prevalence of 16% in the global population (38).

**Figure 2:**
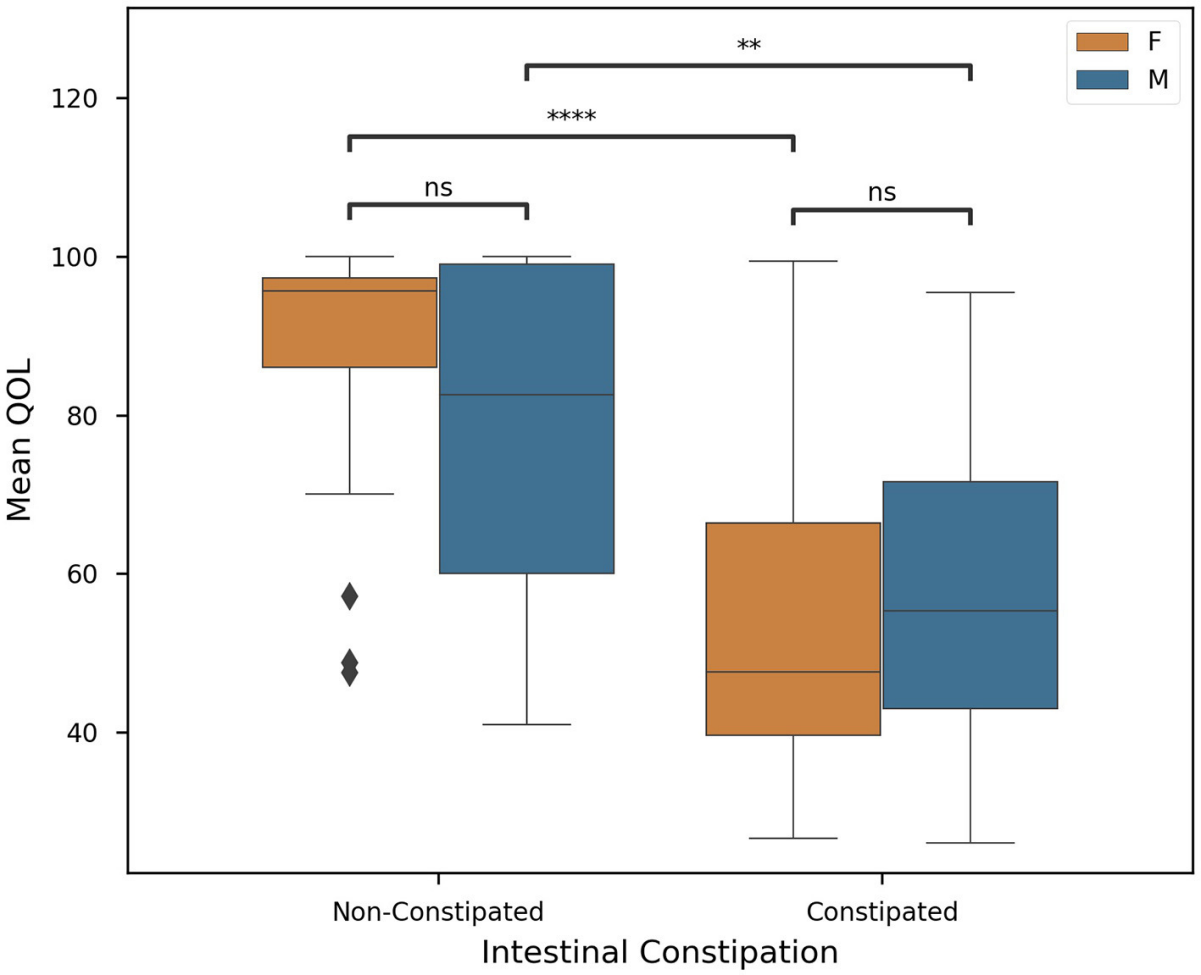
Quality of life in individuals with and without constipation according to sex. G1 presented lower values for all sections of the questionnaire when compared to G2, G3, and G4. In our cohort, we found constipated subjects to have significantly (*p* < 0.0001) lower QOL (56.09 ± 21.86) than those who were non-constipated (82.40 ± 19.35) (*n* = 93, significance was evaluated by two-tailed ANOVA, *p*-value < 0.05). Welch's *t*-tests were employed with Bonferroni corrections for (1) intersex intragroup comparisons and (2) intergroup intrasex comparisons (Key: ns - *p* ≥ 0.05, **p < 0.01, ****p < 0.0001).

According to CPCHILD, the groups differed significantly (*P* < 0.05). G1 presented lower values for all sections of the questionnaire when compared to G2, G3, and G4. In our cohort, we found constipated subjects to have significantly (*P* < 0.0001) lower QOL (56.09 ± 21.86) than those who were non-constipated (82.40 ± 19.35). In Figure 2, this difference is visualized, showing continuity in this hypothesis when stratified by gender. While future studies are required to investigate the full extent of this association, it can be concluded that constipated subjects in our cohort were statistically more likely to present a lower mean quality of life. In Supplementary Figures 1A–E, we plotted each subscore against overall QOL and we observed that higher intensity of discomfort hurt the physical and psychological quality of life.

**Figure 3:**
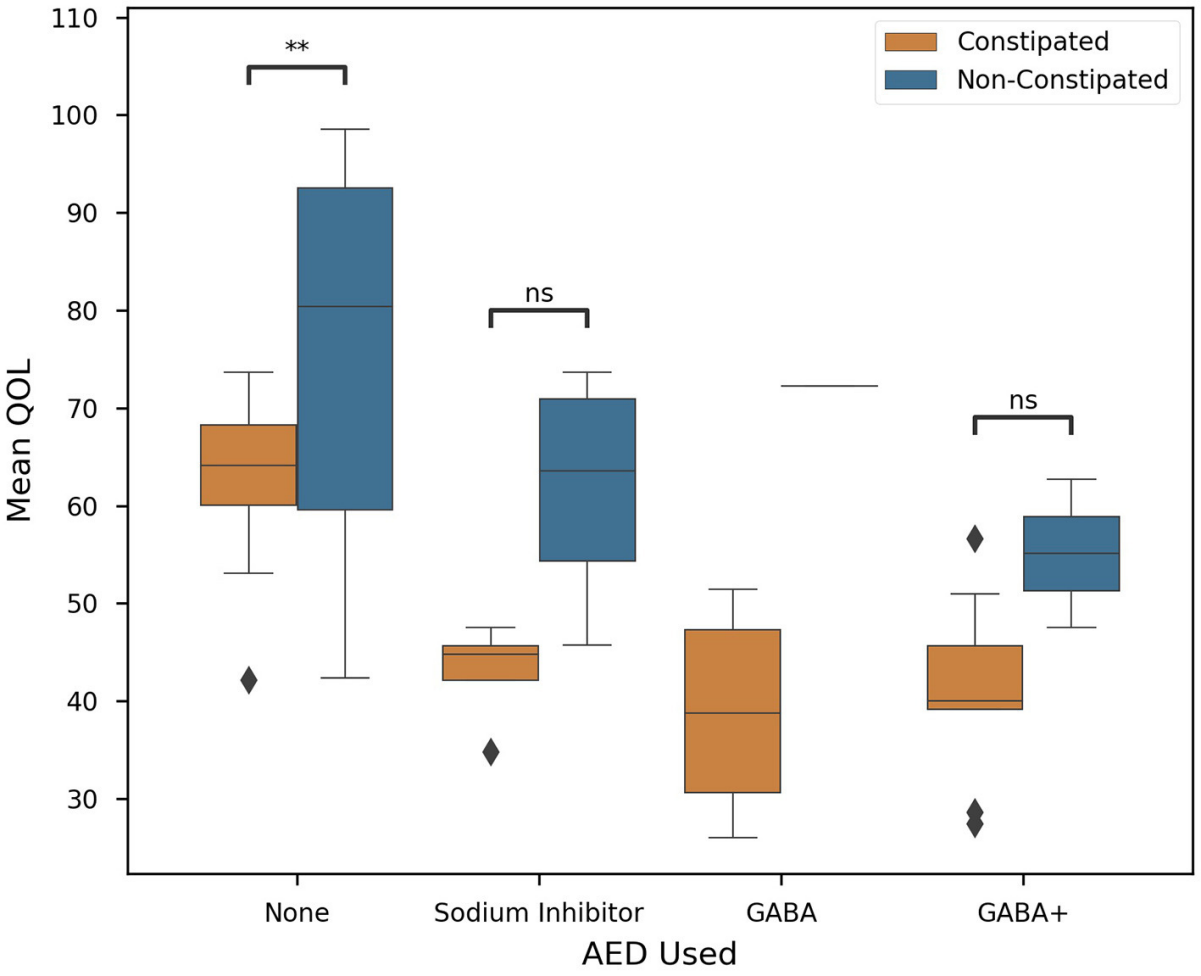
Correlation of clinical factors in CP patients. Constipated subjects are in orange, non-constipated in blue. Colored boxes show interquartile range, black line marks the median, and whiskers show the largest outliers within 150% the interquartile range above the 75th percentile and below the 25th percentile. Remaining outliers are marked with points. Standard box plot comparing AED used and Quality of Life. AED used is significantly correlated with QOL (*P* < 0.0001), *n* = 63, and significance was evaluated by two-tailed ANOVA, *p*-value < 0.05. Where possible (>1 subject), Welch's *t*-tests were employed for each medication on the basis of constipation (Key: ns - *p* ≥ 0.05, **p < 0.01).

The primary factor affecting constipation and QOL in our population was revealed to be the type of medication a patient was prescribed. Here we found that the choice of AED used to combat spasticity was significantly correlated with the QOL (*P* < 0.0001) of our cohort. In Figure 3, AED use is plotted against QOL. We noticed that, in particular, GABA and GABA+ subjects presented lower mean QOL (43.89 ± 12.61) than other groups (78.20 ± 21.39). Meanwhile, sodium inhibitors presented a much higher mean QOL (51.26 ± 12.29). This finding is exceedingly promising, but future investigation is necessary to further explore sodium inhibitors and their effect on QOL.

However, we found that while both AED prescription and intestinal constipation are significant in determining a subject's QOL, ANCOVA revealed that the interaction between these two variables is insignificant (*P* = 0.787). Future studies are required to determine if QOL can be determined solely by the medication or gut health of a patient.

### Salivary Cytokines

In many subjects, the lack of an ability to care for oneself leads to the development of oral and gum disease. If a large number of teeth have bleeding gingiva, there is a high chance of oral disease manifesting itself as gingival inflammation and periodontitis.

**Figure 4:**
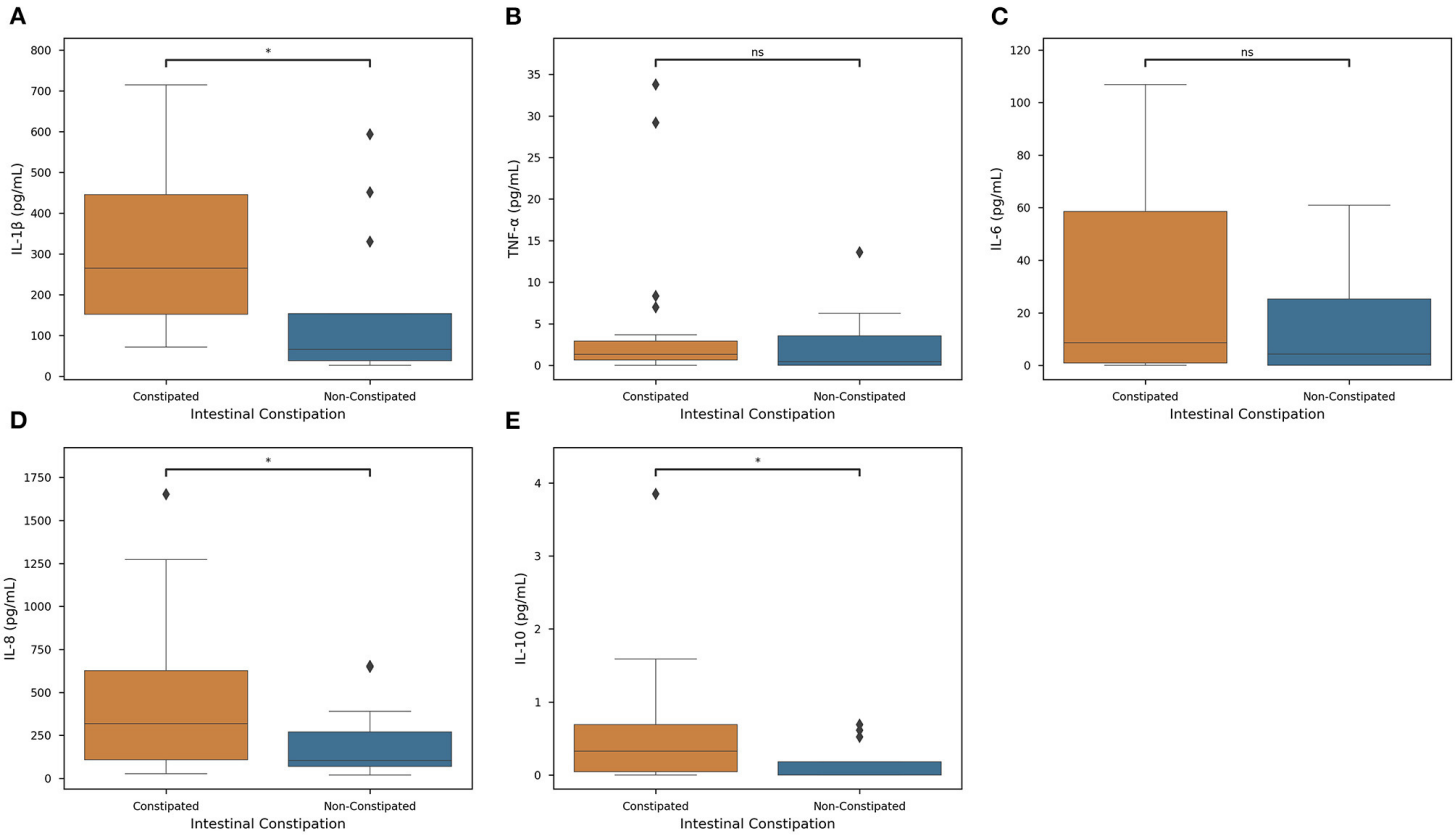
Constipation actions on Inflammatory Cytokine Levels. Distributions of each measured cytokine—**(A)** IL-1β, **(B)** TNF-α, **(C)** IL-6, **(D)** IL-8, and **(E)** IL-10— in constipated subjects (orange) and non-constipated subjects (blue) were mapped. Colored boxes show interquartile range, black line marks the median, and whiskers show the largest outliers within 150% the interquartile range above the 75th percentile and below the 25th percentile (*n* = 63). For each boxplot, Welch's *t*-tests were employed to determine associations between each cytokine and constipation (Key: ns - *p* ≥ 0.05, *p < 0.05).

For a subset of our population, we collected salivary cytokine measures to get a deeper understanding of systemic inflammation and its relationship with constipation and medication. For all cytokines for which data were collected (IL-1β, IL-6, IL-8, IL-10, and TNF-α), mean levels were increased in constipated subjects (Figure 4). Of these cytokines, only IL-8, IL-10, and IL-1β levels were significantly different on the basis of intestinal constipation (*P* < 0.05).

#### Salivary Cytokines as a Predictor of Intestinal Constipation

We first quantified salivary cytokines to predict intestinal constipation yields promising statistical correlations (Figure 4). Of the cytokines measured, IL-1β, IL-8, and IL-10 measures were statistically significant (*P* < 0.05) in determining the presence of intestinal constipation. However, remaining cytokine levels were insignificant in classifying the presence of constipation. Therefore, there exists a specific but significant associative pathway between constipation and cytokine levels.

#### Establishing Oral-Gut Axis for Diagnosis

We found in our cohort that AEDs and intestinal constipation are significantly associated with both GB and one another (*P* < 0.0001). We noticed that, in particular, GABA and GABA+ subjects presented higher GB (39.39 ± 22.86%) than other groups (5.84 ± 9.32%) (Supplementary Figure 2F). In this case, co-measuring AED use and intestinal constipation would prove a more statistically fortified marker for GB than either in isolation.

In the case of gender, we found females to have a mean GB (15.82 ± 22.16%) almost double that of males (8.05 ± 12.04%, Supplementary Figure 3A). Overall mean GB was 12.08 ± 18.27%. In our population, we found gender (*P* < 0.05), CP diagnosis (*P* < 0.0001) and GMFCS classification (*P* < 0.0001) were found to be significantly correlated with GB, while age showed no such significance (Supplementary Figure 3B). Normotypic subjects (those who do not suffer from CP) have markedly lower mean GB 0.6% than any other group, with 2.79% mean hemiplegic GB, 12.19% mean diplegic GB, and 27.11% mean tetraplegic GB (Supplementary Figure 3C).

**Figure 5:**
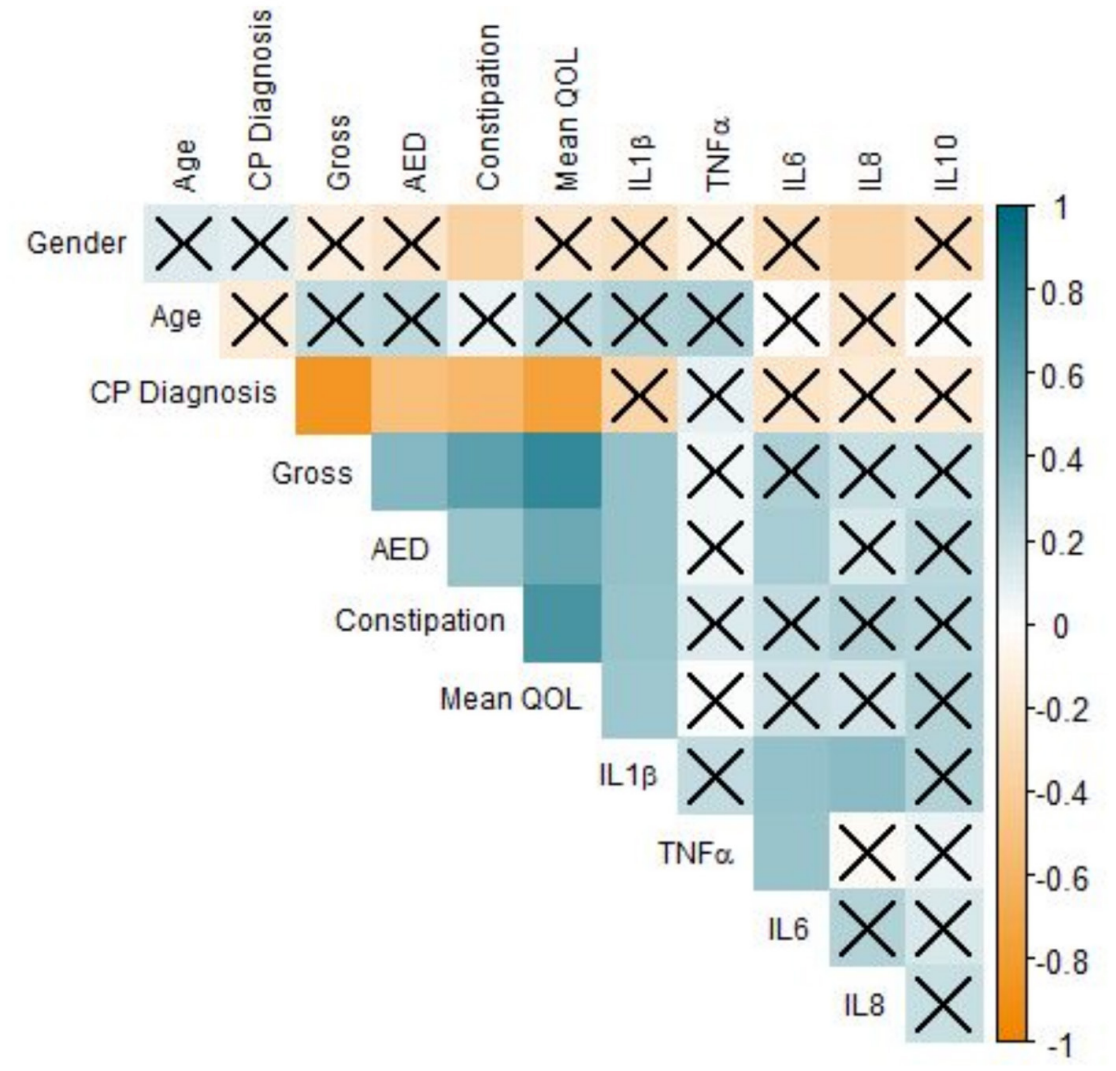
Correlation of clinical factors in CP patients. Correlogram of Pearson's correlation of a subset of clinical factors influencing CP patients. Specificity of our dataset drove subset selection criteria: many factors were removed from this figure but included elsewhere to improve readability. Each axis is labeled by relevant factors to form a lower-triangle correlogram. Each box represents a correlation coefficient of two factors assigned, where size and saturation represents the magnitude of the correlation. Pearson score is represented from −1 (red) to +1 (blue), measuring the magnitude of correlation between the two variables. Boxes overlaid by an X represent associations that are not statistically significant (*P* ≥ 0.05) regardless of the strength of correlation (*n* = 63, significance was evaluated by Pearson's correlation and student's *t*-test, *p*-value < 0.05).

**Figure 6:**
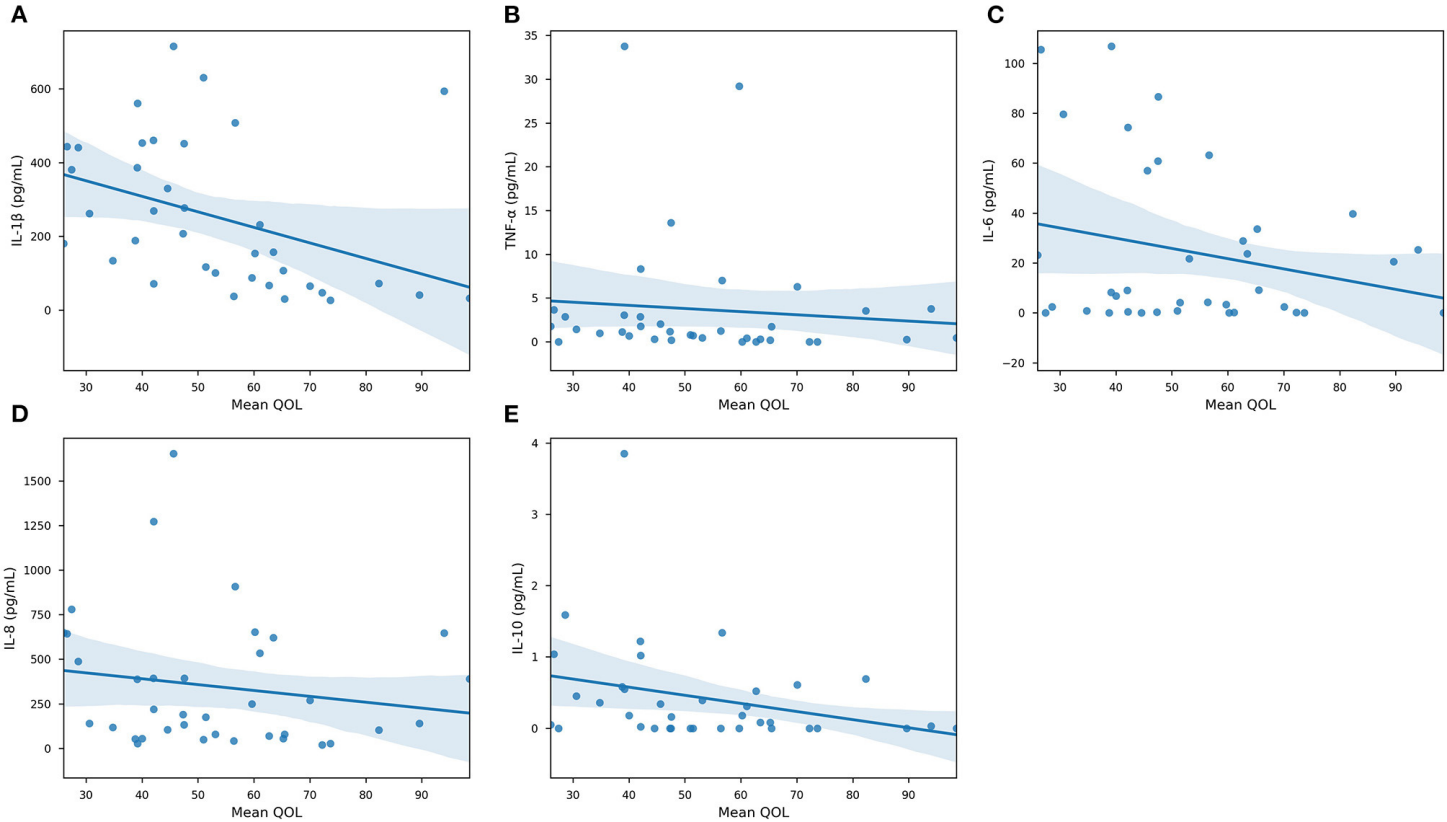
Quality of Life association with specific cytokines. Scatterplots of collected cytokine levels —**(A)** IL-1β, **(B)** TNF-α, **(C)** IL-6, **(D)** IL-8, and **(E)** IL-10—and Quality of Life. Blue line marks the linearly regressed trendline (lm) and the shaded region corresponds to the 95% confidence interval. *P*-values from ANOVA are: IL-1β, *P* < 0.05—TNF-α, *P* = 0.589—IL-6, *P* = 0.156—IL-8, *P* = 0.325—IL-10, *P* = 0.0762 (*n* = 63, significance was evaluated by ANCOVA, *p*-value < 0.05).

Our results demonstrated that IL-1β (*P* < 0.0001), IL-6 (*P* < 0.05), IL-8 (*P* < 0.01), and IL-10 were significantly associated with GB percentage. In Supplementary Figure 2, each cytokine was plotted against GB (%) in an effort to reveal trends that could be fortified by more rigorous statistical methods. In Supplementary Figures 2A,C–E, there is a clear trend indicating that GB was positively correlated (Pearson Correlation) with IL-1β (*R* = 0.720), IL-6 (*R* = 0.343), IL-8 (*R* = 0.425), and IL-10 (*R* = 0.505). In particular, it is important to consider IL-1β, IL-8, and IL-10 in our population, as their levels were significantly correlated with both systemic and oral clinical factors. Interestingly, we found in our population that QOL is more specific, as only IL-1β levels were significant in determining QOL (*P* < 0.05). Furthermore, the AED used by an individual was correlated with IL-1β (*P* < 0.0001) and IL-6 (*P* < 0.05) levels in our salivary cytokine subcohort (Figure 5). While remaining cytokines were not significantly correlated with AEDs, it is important to note that IL-1β was the sole cytokine correlated with QOL. In Figure 6, we plotted each cytokine against QOL. A visual trend connecting IL-1β levels to QOL (Figure 6A) corroborated our statistical findings. IL-10 levels were not significantly correlated with QOL (*P* = 0.076) showing in Figure 6E.

## Discussion

The results provided novel information related to the severity of CP, the presence of inflammation of these subjects, and the consequences of treatment on constipation, oral health, and quality of life. To the best of our knowledge, this is the first study to evaluate the association among salivary inflammation, intestinal constipation, and QOL of CP using the CPCHILD. According to the motor type disorder, subjects with CP are classified as at least one of spastic, dyskinetic (dystonic, choreic, and athetosic), or ataxic. The subjects presented spastic CP, the most prevalent type of motor disorder, with tetraplegia the clinical pattern most prevalent (32.26%) (Figure 1B). Consistent with our results, tetraplegia was also observed in 3,294 children studied in 18 states in the USA (32.9%) (39). Early brain damage triggers complex adaptive neuroplasticity processes involving multiple functional systems. Different factors interact with brain plasticity potentials, influencing the natural history of cerebral palsy in the early years of childhood (40). The later the interdisciplinary intervention, the smaller the gain during brain plasticity, reducing the potential for improvement in the individual's condition.

Here, we observed a shortage of national instruments, which appears to reflect the difficulty of the scientific community to develop tools for assessing the quality of life that apply to the socio-cultural diversity of the country. For all questionnaires revised, only CPCHILD (31) and Cerebral Palsy Quality of Life Questionnaire for Children Child report (CPQol-Child) (41) are specific to CP subjects. CPQoL-Child is the individual's own report since it was mostly used in children with GMFCS level I or II and excluded the individual with any intellectual deficits and lack of an efficient communication system. In this study, the largest number of individuals belonged to GMFCS level IV and V (Figure 1C) and due to the difficulty of communication, their caregivers answered the questionnaire as their proxy. The Cronbach's alpha value estimated for the CPCHILD was 0.879. It was observed that G1 presented a lowest mean score of CPCHILD (51.0 ± 13.1) compared to G2 (71.5 ± 16.7), G3 (88.9 ± 7.5), and G4 (95.5 ± 5.0) (*P* < 0.05) (Table 1). Seventy percent of caregivers of CP subjects in this study had <8 years of study (Table 1). Intellectual capital, represented by education, increases the likelihood of access to information, health-related issues (42) facilitating understanding and recognizing the importance of self-care. A low level of caregiver education influences the child's overall health, represented by self-care of general and oral health (43).

The possible explanation for the lower education level of caregivers for individuals with CP is the “caregiver martyr syndrome” wherein a caregiver neglects their own health, studies, and finances to provide more frequent and consistent care (44). With a lower educational level and fewer paid employment opportunities, there is a direct logical connection linking significantly lower income to caregivers of individuals with CP (Table 1). Caregivers' potentially lower standard of education prevents them from entering the skilled labor market. Thus, they dedicate themselves exclusively to household chores and taking care of the subject with CP (45).

We noticed that great discomfort has been associated with increased motor impairment and other comorbidities, particularly constipation and spasticity (Figure 2). In subjects with minor GMFCS (I, II, and III), the pain was observed during voluntary movements, whereas in subjects with higher GMFCS (IV and V), the pain was directly related to movement performed by the caregiver or therapist (46). Higher intensity of pain and discomfort had a negative effect on the physical and psychological quality of life (Supplementary Figures 1A–E). Communication and social interactions in subjects with CP in GMFCS level V present lower favorably and show large variation (47) (Supplementary Figure 1C). Fairhurst et al. concluded in 2018 that increasing awareness of CP pain and comorbidities, particularly overall health and constipation, can help prevent and treat the disorder more effectively, increasing patients' overall quality of life.

Medication such as AEDs are frequently used for the control of epileptic seizures (48). These drugs can be administered in monotherapy or polytherapy, depending on the response to seizure control (49). Results demonstrated that subjects with a lower quality of life were positively correlated to those who use polytherapeutic GABA treatments (GABA+), while those who use monotherapy, especially sodium channel inhibitors, have a better quality of life when compared with GABA and GABA+ (Figure 3). AEDs have an effect on voltage-dependent modulation of sodium, calcium, and potassium channels, and the GABA is considered the primary inhibitory neurotransmitter not only in the central nervous system but also on enteric nerves (49). However, AEDs aimed to achieve seizure control are usually limited by toxicity and adverse effects that impair an individual's QOL (50) (Figure 3). The subjects taking GABA or GABA association presented a negative correlation with the quality of life and also present intestinal constipation not only for the inhibitory GABA action (51), but may also be related to dysbiosis.

The use of GABA antiepileptic medication and associations to control seizures reduced salivary flow, increased salivary viscosity and gingival bleeding (Supplementary Figure 2F). Thus, the use of medication causes not only constipation but also gingival bleeding and increases the cytokines levels (Supplementary Figures 2A–E) (51). G1 presented significantly higher percentages of subjects with gingivitis compared to all other groups (*P* < 0.001). Additionally, CP subjects using GABA or GABA+ who were constipated had higher levels of GB than those who did not (*P* = 0.006, Table 1).

Accumulation of biofilm acts as a risk factor in the development of the main oral diseases, such as dental caries and periodontal disease. Subjects diagnosed with CP are more susceptible to poor oral hygiene due to neurological impairment and the presence of primitive oral pathological reflexes, facilitating the progression of periodontal disease (52, 53). While a limitation of the study was the lack of evaluation of the oral hygiene index, gingival bleeding index was used as a surrogate of oral inflammation.

Current literature indicates that intestinal disorders play a prominent role in inflammatory responses to neurological conditions (54). This line of evidence is fundamental to identifying the effects of dysbiosis on mucosal inflammation throughout the digestive tract. Significantly higher levels of IL-1β, IL-6, IL-8, and IL-10 were found in constipated subjects with GB from this study (Figure 4), indicating an ongoing inflammatory process and the progression of periodontal disease (55–59). Since the use of these medications cause CP subjects to present a reduced salivary flow rate, an increased value of salivary osmolality, dry mouth, and gingivitis, which is represented by higher levels of inflammatory cytokines in quadriplegics (15). Sodium inhibitors, however, have a less severe correlation with GB (*R*^2^ = 0.684) (Supplementary Figure 2F) but have markedly lower effects on a patient's QOL and they are associated with lower levels of constipation, it can be assumed that sodium inhibitors carry less of a systemic reaction in our study population (Figure 3).

Constipation is common comorbidity described in CP subjects (11), and current literature agrees that constipation is the causal factor of gut dysbiosis (60, 61). Constipation prevalence observed in this study was higher (47.62%). Constipation and intestinal dysbiosis leads to increased mucosal permeability (leaky-gut) (61) of the gut-brain axis (62) increasing serum endotoxin concentration. These endotoxins activate the immune system and promote IL-1β production as observed in our study (Figure 4A) and in individuals with Autism Syndrome Disorder (63). To date, no research has been published that evaluated the effect of dysbiosis on the development of gingivitis in individuals with cerebral palsy.

In addition to GI and oral inflammation, we have observed gender to be an important variable in regard to inflammation. Females presented higher pro-inflammatory cytokine expression when compared to males (Supplementary Figure 3). This gender bias has not been addressed in the literature and will require further studies. The possible explanation for these findings may be related to the phenomenon that CP occurs more frequently in males (64) categorized as GMFCS V (65). Due to this limitation, these subjects require more advanced care, notably oral care and hygiene performed by the caregiver. Perhaps the females were able to perform oral hygiene on their own due to fewer neuromotor limitations, which in turn may have resulted in greater GB. Additionally, hormonal changes in puberty should be examined as a factor for this observed gender dichotomy (66).

Inflammatory biomarkers can be evaluated both in serum (67) or in saliva (8). Blood collection in subjects with CP is a hard task, because these subjects present sympathetic nervous system predominance (68), resulting in vasoconstriction, and consequently making peripheral venous access more difficult. On the other hand, saliva is readily available, and its components are easy to be collected, and that has been used to evaluate salivary parameters in subjects with CP (15), ideal for future longitudinal monitoring.

The comparison between the constipated and non-constipated subjects in this study showed that there was approximately a doubled average production of cytokines favoring the potentiation of inflammation in these individuals (Figures 4A–E). In the presence of dysbiosis, virulence factors are released from pathogenic microorganisms present in the oral cavity, activating host's immune-inflammatory responses (69–71). IL-1β is an inflammatory cytokine associated with innate immune response, inflammation, pathogenesis, and progression of periodontal disease (72, 73). Higher levels of IL-1β (*P* < 0.05) were found in this study's subjects, who were constipated and presented higher values of GB (Figure 4A, Supplementary Figure 2A). Previous studies showed higher levels of IL-1β, IL-6, IL-8 in spastic CP subjects presenting GMCFS level V (15). Significantly higher levels of IL-6, IL-8, and IL-10 were found in constipated subjects with GB (Figures 4C–E; Supplementary Figures 2C–E). These cytokines represent the action of pro-inflammatory and anti-inflammatory cytokines (73), critical biomarkers of periodontal inflammation corresponding to the clinical severity of the disease (56). IL-6 and IL-8 were also described as potential predictors for oral diseases, reinforcing the importance of evaluating the salivary levels of these biomarkers (74). The presence of the chemokine IL-8 induces the secretion of lymphocytes, monocytes, epithelial cells, fibroblasts, tumor cells, bone resorption, and IL-1β, indicating an ongoing inflammatory process and the progression of periodontal disease (57, 58). IL-10 acts as an anti-inflammatory cytokine, inhibiting pro-inflammatory cytokines IL-1 and IL-6 associated with improvements in periodontal clinical parameters (28). However, the degree of mucosal inflammation of constipated CP subjects represented by GB was so high that IL-10 action did not reduce the inflammatory process of dysbiosis (59). Only one study reported the effect of mechanical treatment on gingivitis control measures by cytokines levels, and the results showed that, although reduction of inflammatory process occurs, the levels of IL-1β, IL-6, and IL-8 remain high compared to subjects without CP (8).

These results highlight that prediction of systemic health in CP patients by salivary cytokine monitoring is a reliable approach, yet this field is in its infancy. Novel methodological assays to control for time, type of salivary collection and flow on a longitudinal basis would provide more reliable findings, especially accounting for inter- and intra-individual variations. We were able to demonstrate that not only were there significant differences between gut constipation and oral inflammation with specificity for the type of cytokines, we also demonstrated that specific cytokines significantly correlated with the type of medication. CP inaccessibility and vulnerability to oral care, and consequent development of caries and gum diseases may also impact gut phenotypically via microbiome oral-gut axis. These data does not *per se* indicate microbiome changes, but indicates that gingival bleeding index suggests dysbiosis in host-microbial interactions at the oral mucosal interface, which can, in turn, influence an individual's systemic inflammatory profile. New studies should be developed addressing inflammatory salivary cytokines, oral pathogens correlated with intestinal constipation. While our investigations demonstrated how salivary inflammatory cytokines specifically correlated with gut constipation and quality of life, and it was well-controlled for gender statistical power, limitations were also noted. Dental plaque levels were not recorded. This is an important focus of future studies, specifically dissecting to oral microbes that are modulated by CP environment and medication. We have included gingival bleeding as surrogate to oral host responses to the presence of the biofilm. In future examinations, we aim to look into the multimodal relationship of salivary cytokines, gut constipation as well as gingival bleeding with oral hygiene and dental plaque levels.

In conclusion, the results suggest that the higher levels of inflammatory markers, present in saliva, directly correlated with reduced quality of life, due to excessive gut constipation. Thus, increase of inflammation and gut constipation were directly correlated, impacting quality of life. These are novel relationships in investigating oral-gut-brain axis in investigating the clinical impact of inflammation throughout the human body, and providing reliable information through non-invasive salivary diagnostics.

## Data Availability Statement

The original contributions presented in the study are included in the article/Supplementary Material, further inquiries can be directed to the corresponding author/s.

## Ethics Statement

The studies involving human participants were reviewed and approved by Research Ethics Committee of the Cruzeiro do Sul University-Brazil Platform, São Paulo, Brazil (IRB #2,452,626). Written informed consent to participate in this study was provided by the participants' legal guardian/next of kin.

## Author Contributions

AF, MS, and MF: conceived and designed the experiments and wrote the paper. AF: performed the experiments. AF and RE: analyzed the data. RE: prepared graphs. MF: project administration. All authors: reviewed the manuscript.

## Conflict of Interest

The authors declare that the research was conducted in the absence of any commercial or financial relationships that could be construed as a potential conflict of interest.

## Supplementary Material

Supplementary Figure 1 Effects of CPCHILD Subscores on Overall QOL. Distributions of each individual CPCHILD subscore **(A)** Personal Care; **(B)** Positioning, Transferring, and Mobility; **(C)** Communication and Social Interaction; **(D)** Comfort, Emotions, and Behaviors; **(E)** and Health is measured on the x-axes, while the y-axis marks the average of the remaining subscores. Lines show a trendline of data sets with shaded regions marking the 95% confidence interval (*n* = 93).

Supplementary Figure 2 Gingival Bleeding association with specific cytokines. Scatterplots of collected cytokine levels —**(A)** IL-1β, **(B)** TNF-α, **C)** IL-6, **(D)** IL-8, and **(E)** IL-10—and Gingival Bleeding percentage. Blue line marks the linearly regressed trendline (lm) and the shaded region corresponds to the 95% confidence interval. *P*-values from ANOVA are: IL-1β, *P* < 0.0001—TNF-α, *P* = 0.237—IL-6, *P* < 0.05—IL-8, *P* < 0.01—IL-10, *P* < 0.01. **(F)** Standard box plot comparing AED used and Gingival Bleeding Percentage. AED used is significantly correlated with GB (*P* < 0.0001), *n* = 63, significance was evaluated by ANCOVA, *p*-value < 0.05. Furthermore, both intramedication t-tests were employed in addition to Bonferroni-corrected intermedication Welch's *t*-tests (only constipated subjects) for each medication as compared to control (Key: ns - *p* ≥ 0.05, ^*^*p* < 0.05, ^**^*p* < 0.01, ^***^*p* < 0.001).

Supplementary Figure 3 Factors impacting gingival bleeding. **(A)** Violin plots depicting gingival bleeding patterns stratified by gender. Box plot within the violin represent the interquartile range, and whiskers show the largest outliers within 150% the interquartile range above the 75th percentile and below the 25th percentile. **(B)** Scatter plot demonstrating gingival bleeding in relation to age distribution. Blue line marks trendline, where the shaded region marks the 95% confidence interval. **(C)** Gingival bleeding plotted by Cerebral Palsy Diagnosis. Boxes represent interquartile range, center line represents the median, while whiskers represent 95% confidence interval. The box's color notates the gender, with female being orange and male being blue (*n* = 93). For **(A,C)**, Welch's *t*-tests were employed along gender lines (Key: ns – *p* ≥ 0.05, ^*^*p* < 0.05).

## Abbreviations

CP: Cerebral Palsy
GABA: Gamma-Aminobutyric Acid
GABA+: Gamma-Aminobutyric Acid in association with other medication
CPCHILD: Caregiver Priorities and Child Health Index of Life with Disabilities
QOL: Quality of life
GMFCS: Gross Motor Function Classification System
AED: Antiepileptic drug
AEDs: Antiepileptic drugs
GB: Gingival Bleeding
GI: Gingival Index
BSS: Bristol Stool Scale
CPQoL-Child: Cerebral Palsy Quality of Life Questionnaire for Children Child Report
IL-1β: Interleukin-1 beta
IL-6: Interleukin-6
IL-8: Interleukin-8
CXCL8: Chemokine C-X-C motif ligand 8
IL-10: Interleukin-10
TNF-α: Tumor necrosis factor alpha.
