# Supplementary figures and images for: The Impact of Oral-Gut Inflammation in Cerebral Palsy
Source: Front Immunol. 2021 Feb 25;12:619262. doi: 10.3389/fimmu.2021.619262 (PMC7953843; doi:10.3389/fimmu.2021.619262)

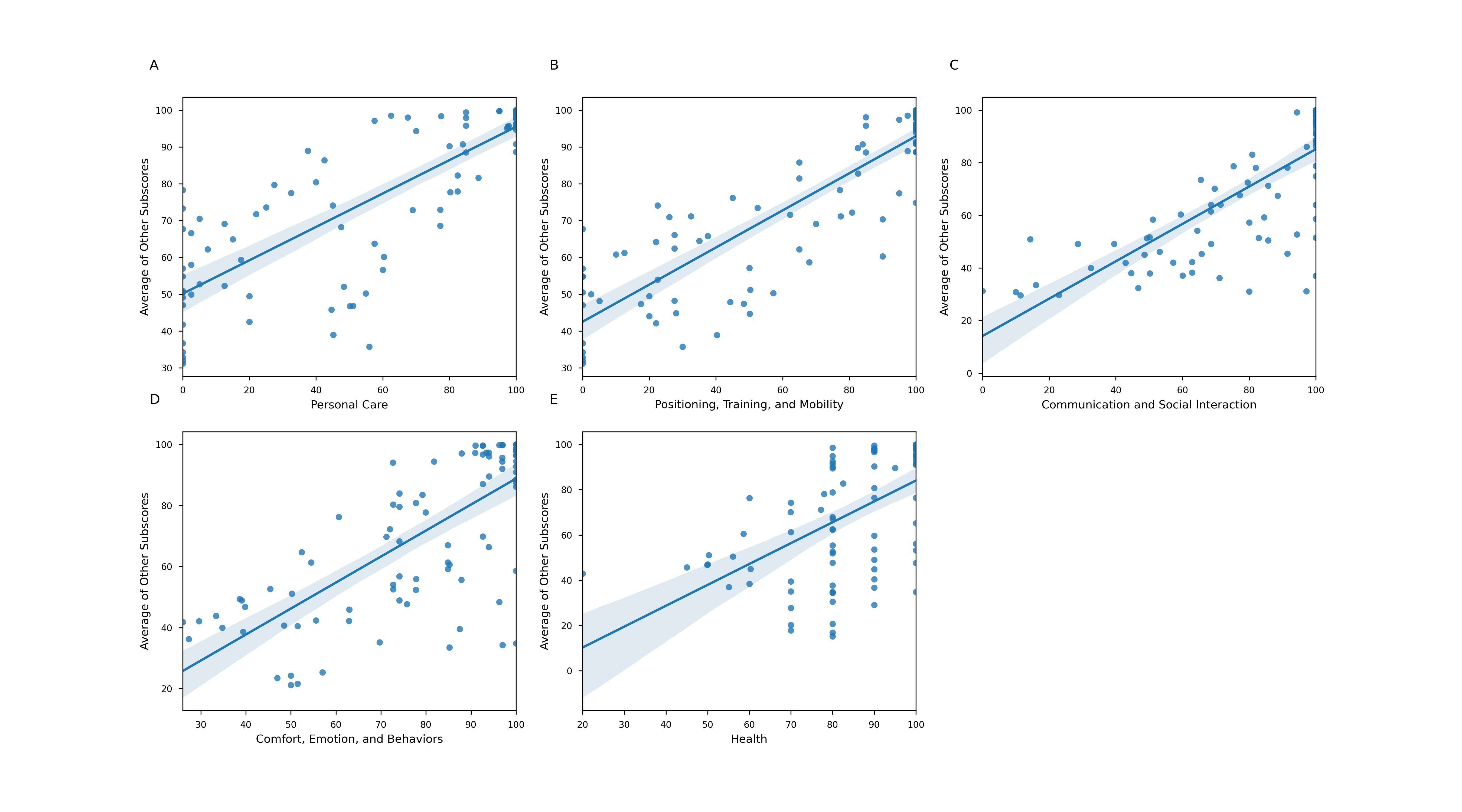

Supplement: Supplementary Figure 1 — Effects of CPCHILD Subscores on Overall QOL. Distributions of each individual CPCHILD subscore (A) Personal Care; (B) Positioning, Transferring, and Mobility; (C) Communication and Social Interaction; (D) Comfort, Emotions, and Behaviors; (E) and Health is measured on the x-axes, while the y-axis marks the average of the remaining subscores. Lines show a trendline of data sets with shaded regions marking the 95% confidence interval (n = 93). [file Image_1.JPEG]

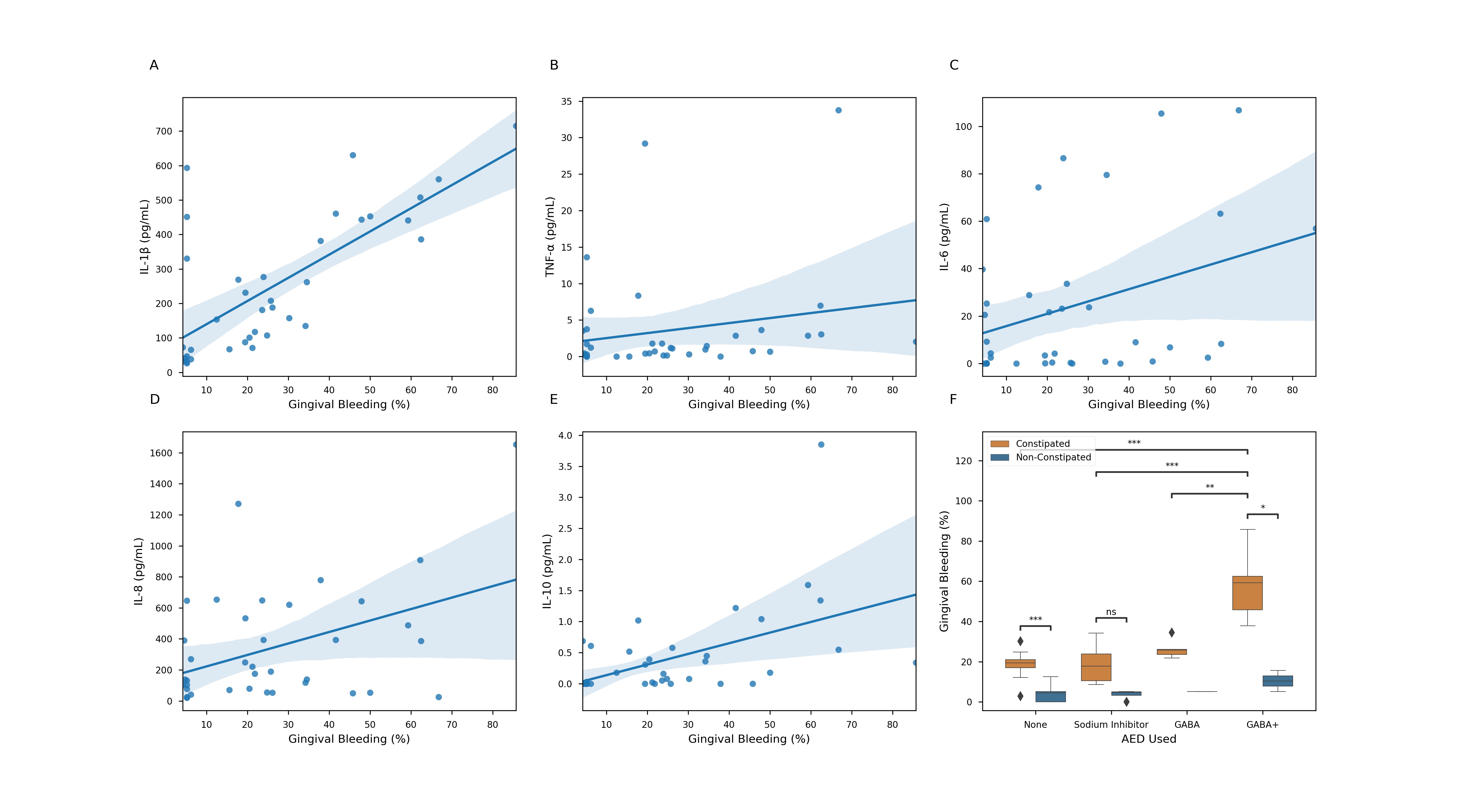

Supplement: Supplementary Figure 2 — Gingival Bleeding association with specific cytokines. Scatterplots of collected cytokine levels —(A) IL-1β, (B) TNF-α, C) IL-6, (D) IL-8, and (E) IL-10—and Gingival Bleeding percentage. Blue line marks the linearly regressed trendline (lm) and the shaded region corresponds to the 95% confidence interval. P-values from ANOVA are: IL-1β, P < 0.0001—TNF-α, P = 0.237—IL-6, P < 0.05—IL-8, P < 0.01—IL-10, P < 0.01. (F) Standard box plot comparing AED used and Gingival Bleeding Percentage. AED used is significantly correlated with GB (P < 0.0001), n = 63, significance was evaluated by ANCOVA, p-value < 0.05. Furthermore, both intramedication t-tests were employed in addition to Bonferroni-corrected intermedication Welch's t-tests (only constipated subjects) for each medication as compared to control (Key: ns - p ≥ 0.05, *p < 0.05, **p < 0.01, ***p < 0.001). [file Image_2.JPEG]

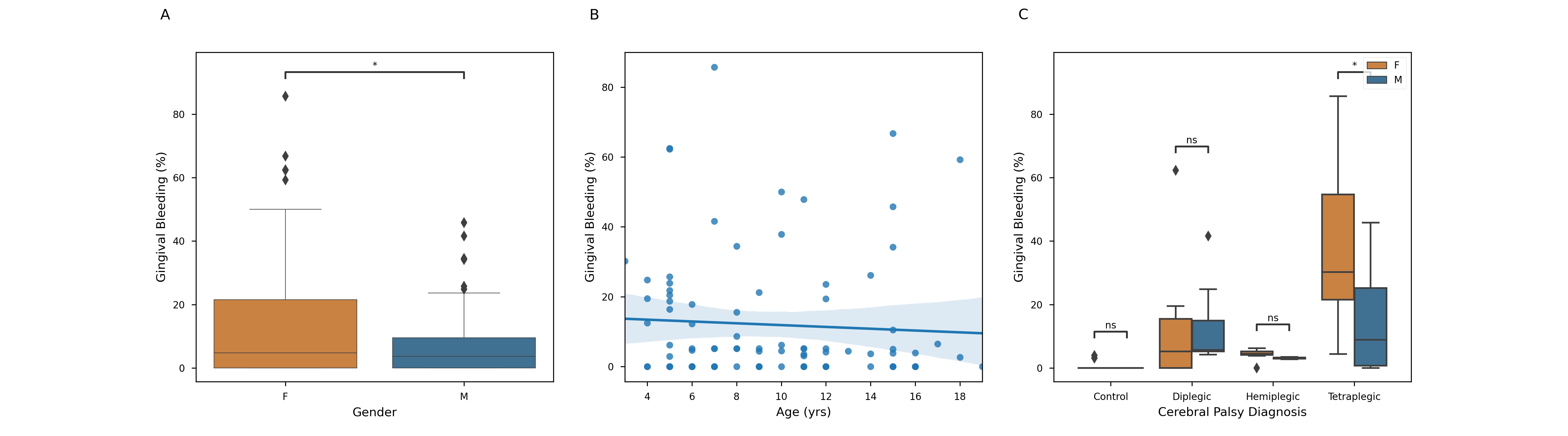

Supplement: Supplementary Figure 3 — Factors impacting gingival bleeding. (A) Violin plots depicting gingival bleeding patterns stratified by gender. Box plot within the violin represent the interquartile range, and whiskers show the largest outliers within 150% the interquartile range above the 75th percentile and below the 25th percentile. (B) Scatter plot demonstrating gingival bleeding in relation to age distribution. Blue line marks trendline, where the shaded region marks the 95% confidence interval. (C) Gingival bleeding plotted by Cerebral Palsy Diagnosis. Boxes represent interquartile range, center line represents the median, while whiskers represent 95% confidence interval. The box's color notates the gender, with female being orange and male being blue (n = 93). For (A,C), Welch's t-tests were employed along gender lines (Key: ns – p ≥ 0.05, *p < 0.05). [file Image_3.JPEG]
